# Stress Management Competency Framework in English policing

**DOI:** 10.1093/occmed/kqz143

**Published:** 2019-11-27

**Authors:** J Houdmont, L Jachens, R Randall, J Colwell, S Gardner

**Affiliations:** 1 Centre for Organizational Health and Development, University of Nottingham, Nottingham, UK; 2 Department of Psychology, Webster University, Geneva, Switzerland; 3 Institute for Work Psychology, University of Sheffield, Sheffield, UK; 4 Devon and Cornwall Police, Exeter, UK; 5 Devon and Cornwall Police Federation, Exeter, UK

**Keywords:** Line manager, police, psychological distress, resilience, Stress Management Competency Framework, work engagement

## Abstract

**Background:**

The UK Health and Safety Executive’s Stress Management Competency Framework and associated questionnaire, the Stress Management Competency Indicator Tool (SMCIT), address line managers’ behaviours across four competency areas. The application in policing remains unexplored.

**Aims:**

This study profiled English police officers’ perception of their line managers’ competencies in the framework areas. The odds of experiencing poor mental wellbeing and work attitudes associated with having a line manager with a development need on each competency area were tested.

**Methods:**

Two hundred and sixty-three police officers completed a survey comprising the SMCIT and measures of psychological distress, resilience and work engagement. Bivariate correlations were calculated to identify patterns of relationships between variables. Binary logistic regression analyses tested the odds of psychological distress caseness, low resilience and low work engagement being associated with officers’ perception of their line manager having a development need on the SMCIT criteria.

**Results:**

Approximately half the participants reported their line manager had a development need on the ‘Managing and Communicating Existing and Future Work’, ‘Managing the Individual Within the Team’ and ‘Reasoning and Managing Difficult Situations’ competencies, and one quarter on the ‘Respectful and Responsible: Managing Emotions and Having Integrity’ competency. Officers’ rating of their line manager having a development need on the four competency areas was associated with up to four-fold elevated odds of each undesirable state.

**Conclusions:**

The framework competency areas are relevant to English policing and offer a basis for stress reduction interventions targeted at line managers’ behaviours.

**Key learning points**

**What is already known about this subject:**

- The behavioural competencies of line managers that effectively manage stress are encompassed in the Stress Management Competency Framework and associated self-report questionnaire, the Stress Management Competency Indicator Tool (SMCIT).
- This study applied the SMCIT to English policing to explore whether it offers a basis to inform stress reduction interventions.

**What this study adds:**

- Half the officers in our study reported their line manager had a development need on three competency areas.
- Having a line manager with a development need on the competency areas was associated with up to four-fold elevated odds of low mental wellbeing and work attitudes.

**What impact this may have on practice or policy:**

- Line managers’ stress management competencies should be targeted for development.
- The Stress Management Competency Framework offers a foundation for the development and evaluation of line manager training activities.
- Work design reviews could assess the extent to which line managers’ roles are clearly defined to facilitate expression of the Framework criteria.

## Introduction

The links between line manager behaviours and subordinates’ stress-related outcomes are well established [1]. The behavioural competencies of line managers that effectively manage stress have been identified [2] and translated into a Stress Management Competency Framework [3]. The UK Health and Safety Executive (HSE) has used this work to make available a self-report questionnaire, the Stress Management Competency Indicator Tool (SMCIT) [4]. In this study, we applied the SMCIT to English policing. 

The SMCIT can be used by line managers to reflect on their own competencies or by subordinates to express their perception of four line manager competencies:

Area 1: *Respectful and Responsible: Managing Emotions and Having Integrity*. Includes sub-competencies of integrity, managing emotions and considerate approach.
Area 2: *Managing and Communicating Existing and Future Work*. Includes sub-competencies of proactive work management, problem solving and participative/empowering.
Area 3: *Managing the Individual Within the Team*. Includes sub-competencies of personally accessible, sociable and empathetic engagement.
Area 4: *Reasoning and Managing Difficult Situations*. Includes sub-competencies of managing conflict, use of organizational resources and taking responsibility for resolving issues.

The research that resulted in the Stress Management Competency Framework and SMCIT was undertaken in five sectors identified by the HSE as ‘high priority’ for stress-related problems (education, finance, local government, central government and healthcare), though the competencies were designed to be applicable across employment sectors. Evidence from use of the SMCIT in Italy has demonstrated its efficacy in supporting the management of stress-related problems in small- and medium-sized enterprises and public sector healthcare and municipality organizations [5–7]. 

Interventions to deal with stress-related problems are needed in English policing [8]. Common mental health problems are prevalent, with rates of psychological distress (anxiety and depression) indicative of minor psychiatric disorder as much as twice that found in the general UK national workforce and three times higher than the English adult population [8,9]. Similarly, the prevalence of high emotional exhaustion (a core dimension of burnout) has been observed as much as double the rate found in the wider population of human service workers [9,10]. A 2016 nationwide study of welfare in English and Welsh policing found that 60% of respondents felt relaxed and 62% felt optimistic about the future never/rarely over the preceding two-week period, with 80% reporting having experienced feelings of stress, low mood or anxiety in the last 12 months; 92% of that number indicating their problems were caused or made worse by work [11]. 

These common mental health problems have attracted growing attention and concern across the policing sector, resulting in a range of initiatives focused on the protection and promotion of mental wellbeing that highlight the important role of the line manager. These include, for example, Public Health England and the College of Policing’s Oscar Kilo online resource (https://oscarkilo.org.uk), the National Police Wellbeing Service that was launched in 2019 following the UK government’s announcement of investment of £7.5 million (https://oscarkilo.org.uk/national-police-wellbeing-service/) and the Police Federation of England and Wales’ Nine-Point Stress Plan [12]. 

What is not yet known is the extent to which line managers in UK policing typically display competencies known to protect and promote mental wellbeing in others. Moreover, the links between these competencies and mental wellbeing and work attitudes in the policing context remain unclear. Low levels of the competencies and linkages between these and undesirable mental wellbeing states would indicate that the selection and development of line managers could be fruitful avenues for intervention. This study offers a preliminary examination of the applicability of the Stress Management Competency Framework and SMCIT in English policing. Specifically, it aims to profile line manager stress management competencies in accordance with the framework and examine the strength of association between subordinates’ perception of their line manager’s competencies and their psychological distress, resilience and work engagement. 

## Methods

Police officers in two command units of an English county force were invited to complete an online survey in January 2019. Eligible officers were made aware of the survey through intranet and email communications that included a hyperlink to the survey and endorsement from a Chief Superintendent. Participation in the study was voluntary and anonymous. The Faculty of Medicine and Health Sciences Research Ethics Committee at the University of Nottingham granted ethical approval (ref: 44-1807).

Respondents rated their line manager on the four Stress Management Competency Framework areas using the 36-item version of the SMCIT that assesses each area via nine items [7]. Sample items include ‘My line manager…’ ‘does not speak about team members behind their backs’ (Area 1: Respectful and Responsible: Managing Emotions and Having Integrity), ‘when necessary will stop additional work being passed on to me’ (Area 2: Managing and Communicating Existing and Future Work), ‘is available to talk when needed’ (Area 3: Managing the Individual Within the Team) and ‘deals objectively with employee conflicts’ (Area 4: Reasoning and Managing Difficult Situations). Each item is scored on a five-point scale of (1) strongly disagree, (2) disagree, (3) slightly agree, (4) agree and (5) strongly agree, with a sum score calculated for each competency area following reverse scoring of negatively framed items. The sum score for each area is converted into a percentage of the maximum possible score (which is 45), with higher scores indicating higher competency displays. The HSE advises that scores of ≤75% (≤33) indicate the line manager has a development need and would benefit from developing their competencies in order to be more effective at preventing and reducing stress in their team. Scores of 76–89% (34–40) suggest the line manager is reasonable and shows good awareness of the behaviours needed to effectively prevent and reduce stress in others though may benefit from some further development. Scores of ≥90% (≥41) indicate the line manager is effective in their demonstration of the competency [4]. 

Psychological distress was measured using the 12-item General Health Questionnaire (GHQ-12) [13]. Responses to the first six items (e.g. ‘[over the past few weeks have you] been able to concentrate on whatever you are doing?’) are given on a four-point scale of ‘more so than usual’, ‘same as usual’, ‘less than usual’ and ‘much less than usual’, while responses to the remaining items (e.g. ‘[over the past few weeks have you] lost much sleep over worry?’) are given on a scale of ‘not at all’, ‘no more than usual’, ‘rather more than usual’ and ‘much more than usual’. We used the GHQ scoring method (0–0–1–1) with responses summed to a global score ranging from 0 to 12 and dichotomized into non-distressed (GHQ score 0–3) and distressed (GHQ score 4–12). The 3/4 threshold is the most accurate for identifying likely cases of minor psychiatric morbidity in the general UK working population [14,15] and has been widely used to differentiate between likely cases of minor psychiatric disorder and non-cases [16,17]. 

Resilience—a person’s ability to deal with and bounce back from adversity—was measured using the Brief Resilience Scale (BRS) [18]. This measure was selected for two reasons. First, the BRS assesses the construct’s core components of recovery, resistance, adaptation, and thriving and not its antecedents [19]. Second, its brevity helps prevent the overall questionnaire from becoming too lengthy. A sample item is ‘I tend to bounce back quickly after hard times’, with responses given on a five-point scale of (1) strongly disagree, (2) disagree, (3) neutral, (4) agree and (5) strongly agree. An overall mean score was generated after reverse scoring of negatively framed items and dichotomized using the median split (score of 3.50) to generate low and high resilience classifications. 

Work engagement was assessed using the Ultra-Short Utrecht Work Engagement Scale (UWES-3) [20]. The three-item version of the scale was selected in order to minimize the assessment burden on participants. Reliability and validity are comparable to that of the widely used nine-item version [20]. The UWES-3 measures each of three dimensions of work engagement via a single-item: ‘At my work I feel bursting with energy’ (vigour), ‘I am enthusiastic about my job’ (dedication) and ‘I am immersed in my work’ (absorption), with responses given on a seven-point scale of (0) never, (1) almost never, (2) rarely, (3) sometimes, (4) often, (5) very often and (6) always. An overall mean score was calculated and dichotomized using the median split (score of 3.33) to generate low and high work engagement classifications. 

We performed analyses using IBM SPSS V.24. Descriptive statistics and reliability coefficients were generated and bivariate correlations calculated to highlight patterns of relationships between variables. To examine the likelihood of psychological distress caseness, low resilience and low work engagement being associated with having a line manager identified as either reasonable or having a development need on each competency area, we used binary logistic regression to generate odds ratios (ORs) with 95% confidence intervals (CIs). For each OR, the reference category was the presumed least hazardous arrangement, i.e., having a line manager identified as effective. Crude ORs were calculated in addition to a model that adjusted for potentially confounding socio- and occupational-demographic variables (age, gender, rank, role). Statistical significance was defined as *P* < 0.05 throughout.

## Results

Two hundred and sixty-three officers completed the survey (22% response rate). Respondents’ socio- and occupational-demographic characteristics are shown in Table 1. All scale reliabilities exceeded the commonly held minimum threshold for acceptable internal consistency of 0.70 [21]. The proportion of respondents reporting their line manager as having a development need on the four competency areas was high: Respectful and Responsible: Managing Emotions and Having Integrity (26%), Managing and Communicating Existing and Future Work (52%), Managing the Individual Within the Team (58%), and Reasoning and Managing Difficult Situations (46%). Descriptive statistics, scale reliabilities and bivariate correlations between scale variables are shown in Table 2. Correlation analyses identified significant associations of small effect size (*r* = 0.10–0.29) [22] in the expected direction between the four line manager competency areas and the target variables, with higher competency levels associated with lower psychological distress, higher resilience and higher work engagement. 

**Table 1.** Participant characteristics

	*N* (%)
Gender	
Male	192 (73)
Female	67 (25)
Not specified	4 (2)
Age	
≤29	24 (9)
30–39	67 (25)
40–49	95 (36)
50–59	65 (25)
≥60	10 (4)
Not specified	2 (1)
Rank	
PCSO	54 (21)
Constable	151 (57)
Sergeant	38 (14)
Inspector/chief inspector	12 (5)
Not specified	8 (3)
Role	
Local investigation	15 (6)
Neighbourhood policing	90 (34)
Response	135 (51)
Other	19 (7)
Not specified	4 (2)

**Table 2.** Descriptive statistics, scale reliabilities and correlations between study variables

	*M*	SD	Range	α	1	2	3	4	5	6
1. Psychological distress	3.80	4.00	0–12	0.93						
2. Resilience	3.42	0.80	1–5	0.90	−0.45**					
3. Work engagement	3.34	1.07	0–6	0.77	−0.35**	0.40**				
4. Competency area 1: Respectful and Responsible: Managing Emotions and Having Integrity	80.45	14.72	22–100	0.91	−0.23**	0.26**	0.18**			
5. Competency area 2: Managing and Communicating Existing and Future Work	71.66	15.12	20–100	0.93	−0.23**	0.25**	0.25**	0.83**		
6. Competency area 3: Managing the Individual Within the Team	71.21	16.03	24–100	0.93	−0.13*	0.21**	0.17**	0.74**	0.77**	
7. Competency area 4: Reasoning and Managing Difficult Situations	71.97	15.62	20–100	0.96	−0.19**	0.19**	0.21**	0.74**	0.80**	0.78**

α = Cronbach’s alpha coefficient.

*P < 0.05, **P < 0.01.

In binary logistic regression analysis (Table 3), after adjustment for socio- and occupational-demographic characteristics, a report of having a line manager with a development need on the first of the Stress Management Competency Framework areas (Respectful and Responsible: Managing Emotions and Having Integrity) was associated with significantly increased odds of psychological distress caseness (OR 3.96, 95% CI 1.84–8.52) and low resilience (OR 4.64, 95% CI 2.16–9.97). For the second competency area (Managing and Communicating Existing and Future Work), a report of having a line manager with a development need was associated with significantly elevated odds of psychological distress caseness (OR 3.06, 95% CI 1.10–8.54) and low work engagement (OR 4.42, 95% CI 1.37–14.21). A report of a line manager with a development need on the third competency area (Managing the Individual Within the Team) was associated with significantly elevated odds of psychological distress caseness (OR 2.78, 95% CI 1.13–6.81), low resilience (OR 3.20, 95% CI 1.35–7.61) and low work engagement (OR 2.98, 95% CI 1.21–7.38). Finally, a report of a line manager with a development need on the fourth competency area (Reasoning and Managing Difficult Situations) was associated with significantly elevated odds of low resilience (OR 2.84, 95% CI 1.00–8.07).

**Table 3.** Binary logistic regression of line manager stress management competencies in relation to psychological health and work attitudes

Competency areas	Psychological distress (case)			Resilience (low)			Work engagement (low)		
*N* (%)	*N* (%)	OR (95% CI)	AOR (95% CI)	*N* (%)	OR (95% CI)	AOR (95% CI)	*N* (%)	OR (95% CI)	AOR (95% CI)
Competency area 1: Respectful and Responsible: Managing Emotions and Having Integrity									
Effective 76/241 (31)	27/75 (36)	Ref.	Ref.	22/75 (29)	Ref.	Ref.	27/76 (36)	Ref.	Ref.
Reasonable 103/241 (43)	43/102 (42)	1.30 (0.71–2.39)	1.49 (0.77–2.88)	56/103 (54)	**2.87 (1.53**–**5.39)**	**3.08 (1.59**–**5.99)**	45/103 (44)	1.41 (0.77–2.59)	1.71 (0.90–3.28)
Development need 62/241 (26)	39/61 (64)	**3.15 (1.56**–**6.37)**	**3.96 (1.84**–**8.52)**	39/62 (63)	**4.09 (2.00**–**8.36)**	**4.64 (2.16**–**9.97)**	29/62 (47)	1.60 (0.80–3.17)	1.77 (0.86–3.66)
Competency area 2: Managing and Communicating Existing and Future Work									
Effective 24/236 (10)	7/24 (29)	Ref.	Ref.	9/24 (38)	Ref.	Ref.	5/24 (21)	Ref.	Ref.
Reasonable 89/236 (38)	34/89 (38)	1.50 (0.56–3.99)	1.80 (0.63–5.16)	36/88 (41)	1.15 (0.46–2.92)	1.23 (0.46–3.30)	36/89 (40)	2.58 (0.88–7.54)	3.26 (0.99–10.71)
Development need 123/236 (52)	62/121 (51)	**2.55 (1.00**–**6.60)**	**3.06 (1.10**–**8.54)**	67/122 (55)	2.03 (0.83–4.99)	2.12 (0.81–5.54)	58/123 (47)	**3.39 (1.19**–**9.66)**	**4.42 (1.37**–**14.21)**
Competency area 3: Managing the Individual Within the Team									
Effective 31/240 (13)	8/31 (26)	Ref.	Ref.	9/31 (29)	Ref.	Ref.	8/31 (26)	Ref.	Ref.
Reasonable 69/240 (29)	33/69 (48)	**2.64 (1.04**–**6.70)**	2.41 (0.92–6.31)	32/68 (47)	2.17 (0.88–5.40)	2.13 (0.84–5.44)	28/69 (41)	1.96 (0.77–5.01)	2.23 (0.84–5.94)
Development need 140/240 (58)	65/137 (47)	**2.60 (1.09**–**6.21)**	**2.78 (1.13**–**6.81)**	79/140 (56)	**3.17 (1.36**–**7.37)**	**3.20 (1.35**–**7.61)**	66/140 (47)	**2.56 (1.07**–**6.12)**	**2.98 (1.21**–**7.38)**
Competency area 4: Reasoning and Managing Difficult Situations									
Effective 23/230 (10)	10/23 (43)	Ref.	Ref.	7/23 (30)	Ref.	Ref.	7/23 (30)	Ref.	Ref.
Reasonable 102/230 (44)	41/101 (41)	0.89 (0.36–2.22)	0.93 (0.35–2.48)	50/101 (50)	2.24 (0.85–5.91)	2.65 (0.93–7.55)	38/102 (37)	1.36 (0.51–3.60)	1.27 (0.45–3.57)
Development need 105/230 (46)	58/103 (56)	1.68 (0.67–4.17)	1.83 (0.69–4.87)	55/105 (52)	2.51 (0.96–6.61	**2.84 (1.00**–**8.07)**	52/105 (50)	2.24 (0.85–5.90)	2.11 (0.75–5.90)

Significant findings in bold. AOR adjusted for socio-demographic characteristics (age, gender) and occupational-demographic characteristics (rank, role). AOR, adjusted odds ratio; Ref., reference category.

## Discussion

In our sample of English police officers, approximately half the participants reported their line manager had a development need on the Managing and Communicating Existing and Future Work, Managing the Individual Within the Team, and Reasoning and Managing Difficult Situations Stress Management Competency Framework areas, while one quarter reported their line manager had a development need on the Respectful and Responsible: Managing Emotions and Having Integrity competency area. Officers’ reports of working with a line manager with a development need on each of the four competency areas were associated with elevated odds of psychological distress, low resilience and low work engagement. Those that reported their line manager had a development need on the Respectful and Responsible: Managing Emotions and Having Integrity competency area had four-fold increased odds of psychological distress caseness and low resilience. A development need on the Managing and Communicating Existing and Future Work competency area was associated with three-fold increased odds of psychological distress caseness and four-fold increased odds of low work engagement. A development need on the Managing the Individual Within the Team competency area was associated with three-fold increased odds for all three outcomes. Finally, a development need on the Reasoning and Managing Difficult Situations competency area was associated with three-fold increased odds of low resilience. 

The results of this study should be interpreted in light of its limitations. The cross-sectional design hampered the interpretation of causality; on the basis of these findings it is not possible to conclude definitively that line manager competencies precede psychological health and work attitudes as associations could result from reverse causality or reciprocal relationships. Furthermore, officers experiencing poor mental health might require additional support from their line manager and report dissatisfaction with their line manager’s competencies if they perceive that this is not received. The design also prevents conclusions on whether the relationship between line manager competencies and subordinates’ mental health and work attitudes is direct or mediated through the impact of line manager behaviours on working conditions [1]. This preliminary study involved police officers drawn from a restricted set of occupational roles within a single force, potentially limiting the findings’ generalizability. These concerns could be addressed through future research involving a large-scale nationally representative sample and longitudinal design. The relatively low response rate prevents us from discounting the possibility of non-response bias; however, this possibility is mitigated owing to participants broadly reflecting the population from which they were drawn on key socio- and occupational-demographic characteristics. Nevertheless, the possibility remains that those with pre-existing common mental health conditions might have been more or less likely to self-select into the study. 

The Stress Management Competency Framework and accompanying SMCIT “offers an evidence-based practical checklist to inform the assessment of management skills, training and development” (p. 308) [23] that the current study has shown to be applicable to policing in England. Our findings provide useful benchmark data and indicate that in the force in which the study was conducted there exists an imperative for training and development in relation to line managers’ stress management competencies, with the Stress Management Competency Framework offering a foundation for such activities. The findings further suggest that a work design review would help to assess the extent to which line managers’ roles are clearly defined to encapsulate and facilitate expression of these competencies. Associated with this, line managers’ views should be elicited on the extent to which these competencies are central to their role and the degree of scope available to enact them within daily activities. It would also be useful to explore the views of subordinates on their expectations of line managers in relation to the four competency areas; it is possible that some of the generic Stress Management Competency Framework areas might not be viewed as relevant or necessary in policing while other sector- and role-specific competencies might be identified as important. 

Given that our sample comprised officers in a range of roles and career stages it is probable that the competency levels and associations between line manager competencies and subordinates’ mental health and work attitudes observed are indicative of the wider national picture for policing in England. An imperative exists to establish the extent to which that is the case. Moreover, training interventions based on the generic Stress Management Competency Framework while tailored to the policing context ought to be developed and evaluated. 

Our findings also suggest that the Framework might be usefully integrated into performance management processes for those with people management responsibility and guide the selection and assessment of future policing leaders. This could be beneficial for “where managers are selected, developed and rewarded for showing competence in managing stress in their employees, the relevant behaviours should become the norm, resulting in enhanced wellbeing for employees” (p. 313) [23]. 

A small number of Australian policing studies have demonstrated that stress management training for line managers can generate reductions in stress-related problems in subordinates. For example, Biggs and colleagues [24] found that a leadership development intervention had a positive effect at 7-month follow-up on subordinates’ perceptions of supportiveness of the work culture, personal alignment with the strategic priorities of the organization, work engagement and job satisfaction. Results from these studies are not universally positive; Biggs and colleagues observed no significant gains for job demands, supportive leadership, psychological strain or turnover intentions, which is disappointing given the time-intensive nature of the 5-day leadership development intervention. A further Australian policing study involving supportive leadership training for station leaders found no improvement in any of the measured outcomes among subordinates, a situation ascribed to practical difficulties with implementation of the intervention and the possibility that the period between training and outcome assessment was too short for behaviours to have become embedded and had an impact [25]. Studies such as these suggest that line management training may boost the wellbeing of police officers and that practical and context-specific issues need to be accounted for in design and implementation, highlighting the development of sector-specific tailored interventions as a potentially fruitful course of action. Our findings suggest that in the English policing context line manager training informed by the HSE Stress Management Competency Framework holds the potential to produce gains for officers’ mental health and work attitudes, and by extension performance.

## Funding

Financial support for this research was received from Devon and Cornwall Police and Devon and Cornwall Police Federation.

## Competing interests

This study was undertaken in, and involved officers of, the Devon and Cornwall Constabulary. J.C. is in a full-time paid position at Devon and Cornwall Police. S.G. is in a full-time paid position at Devon and Cornwall Police Federation. J.H., L.J. and R.R. have no conflicts of interest to declare.

## References

[1] Skakon J, Nielsen K, Borg V, Guzman J Are leaders’ well-being, behaviours and style associated with the affective well-being of their employees? A systematic review of three decades of research. Work Stress 2010;24:107–139.

[2] Yarker J, Donaldson-Feilder E, Lewis R, Flaxman P *Management Competencies for Preventing and Reducing Stress at Work. Identifying and Developing the Management Behaviours Necessary to Implement the HSE Management Standards* Report Number RR553. 2007 http://www.hse.gov.uk/research/rrpdf/rr553.pdf (17 April 2019, date last accessed).

[3] Yarker J, Lewis R, Donaldson-Feilder E *Management Competencies for Preventing and Reducing Stress at Work: Identifying and Developing the Management Behaviours Necessary to Implement the HSE Management Standards: Phase Two* Report Number RR633. 2008 http://www.hse.gov.uk/research/rrpdf/rr633.pdf (17 April 2019, date last accessed).

[4] Health and Safety Executive. *Stress Management Competency Indicator Tool*. 2009 http://www.hse.gov.uk/stress/mcit.htm (17 April 2019, date last accessed).

[5] Toderi S, Balducci B Stress-preventive management competencies, psychosocial work environments, and affective wellbeing: a multi-level, multisource investigation. Int J Environ Res Public Health 2018;15:397. 10.3390/ijerph15030397 PMC5876942 29495360

[6] Toderi S, Gaggia A, Balducci C, Sarchielli G Reducing psychosocial risks through supervisors’ development: a contribution for a brief version of the ‘Stress Management Competency Indicator Tool’. Sci Total Environ 2015; 518–519:345–351. 10.1016/j.scitotenv.2015.02.082 25770947

[7] Toderi S, Sarchielli G Psychometric properties of a 36-item version of the ‘Stress Management Competency Indicator Tool’. Int J Environ Res Public Health 2016;13:1086. 10.3390/ijerph13111086 PMC5129296 27827940

[8] Houdmont J Stressors in police work and their consequences. In: Burke R., ed. Stress in Policing: Sources, Consequences, and Interventions. London: Routledge, 2017; 51–65.

[9] Houdmont J, Randall R Working hours and common mental disorders in English police officers. Occup Med (Lond) 2016;66:713–718. 27852878 10.1093/occmed/kqw166

[10] Houdmont J *Custody Officers’ Stress-Related Working Conditions: Relations With Health and Organisational Effectiveness* Research Report for the Sergeants’ Central Committee of the Police Federation of England and Wales 2014.

[11] Houdmont J, Elliott-Davies M *Police Federation 2016 Demand, Capacity, and Welfare Survey: Initial Descriptive Findings Report*. Research Report for the Police Federation of England and Wales 2016 http://www.polfed.org/documents/Welfare%20Survey%202016%20-%20PFEW%20Descriptive%20Results%20Report%20v3.0.pdf (17 April 2019, date last accessed).

[12] Police Federation of England and Wales. *Nine-Point Stress Plan* 2017 http://www.polfed.org/newsroom/4982.aspx (17 April 2019, date last accessed).

[13] Goldberg D, Williams P. User’s Guide to the General Health Questionnaire. Windsor, UK: NFER-Nelson, 1988.

[14] Goldberg DP, Gater R, Sartorius N et al. The validity of two versions of the GHQ in the WHO study of mental illness in general health care. Psychol Med 1997;27:191–197. 9122299 10.1017/s0033291796004242

[15] Hardy GE, Shapiro DA, Haynes CE, et al. Validation of the General Health Questionnaire-12 using a sample of employees from the healthcare services. Psychol Assess 1999;11:159–165.

[16] Goodwin L, Ben-Zion I, Fear NT, Hotopf M, Stansfeld SA, Wessely S Are reports of psychological stress higher in occupational studies? A systematic review across occupational and population based studies. PLoS ONE 2013;8:e78693. 24223840 10.1371/journal.pone.0078693 PMC3817075

[17] Murphy H, Lloyd K Civil conflict in Northern Ireland and the prevalence of psychiatric disturbance across the United Kingdom: a population study using the British Household Panel Survey and the Northern Ireland Household Panel Survey. Int J Soc Psychiatry 2007;53:397–407. 18018662 10.1177/0020764007078340

[18] Smith BW, Dalen J, Wiggins K, Tooley E, Christopher P, Bernard J The Brief Resilience Scale: assessing the ability to bounce back. Int J Behav Med 2008;15:194–200. 18696313 10.1080/10705500802222972

[19] Fletcher D, Sarkar M Psychological resilience: a review and critique of definitions, concepts, and theory. Eur Psychol 2013;18:12–23.

[20] Schaufeli W, Shimazu A, Hakanen J, Salanova M, De Witte H An Ultra-Short Measure for Work Engagement: the UWES-3 validation across five countries. Eur J Psychol Assess 2019;35:577–591.

[21] Nunnally J, Bernstein I. Psychometric Theory. 3rd edn. New York: McGraw-Hill, 1994.

[22] Cohen J. Statistical Power and Analysis for the Behavioral Sciences. 2nd edn. Hillsdale, NJ: Lawrence Erlbaum, 1988.

[23] Lewis R, Yarker J, Donaldson-Feilder E, Flaxman P, Munir F Using a competency-based approach to identify the management behaviours required to manage workplace stress in nursing: a critical incident study. Int J Nurs Stud 2010;47:307–313. 19699476 10.1016/j.ijnurstu.2009.07.004

[24] Biggs A, Brough P, Barbour J Enhancing work-related attitudes and work engagement: a quasi-experimental study of the impact of an organizational intervention. Int J Stress Manag 2014;21:43–68.

[25] LaMontagne A *An Integrated Workplace Mental Health Intervention in Victoria Police: Results of a Cluster-Randomised Trial* Research Report for WorkSafe Victoria, through the Institute for Safety, Compensation, and Recovery Research. 2017 https://research.iscrr.com.au/__data/assets/pdf_file/0020/1024715/an-integrated-workplace-mental-health-intervention-in-Victoria-Police.pdf (17 April 2019, date last accessed).

